# RORγ drives non-small cell lung cancer progression by upregulating the NGF signaling

**DOI:** 10.1186/s12931-026-03523-7

**Published:** 2026-01-31

**Authors:** Yechun Zeng, Guodi Cai, Jian Zhang, Zhenhua Zhang, Wenxin Yin, Tianmiao Ou, Meng Xu, Jing Li, Zhanfang Kang, Junguo Bu, Junjian Wang, Jie Huang, Weineng Feng

**Affiliations:** 1 National-Local Joint Engineering Laboratory of Druggability and New Drugs Evaluation, School of Pharmaceutical Sciences, Sun Yat-Sen University, Guangzhou, Guangdong 510006 China; 2 Thoracic Surgery Department, The Third Affiliated Hospital of Sun Yat-sen University, No. 600, Tianhe Road, Tianhe District, Guangzhou, Guangdong 510630 China; 3 School of Life Sciences and Biopharmaceutics, Guangdong Pharmaceutical University, Guangzhou, Guangdong 510006 China; 4 Foshan Key Laboratory of Precision Therapy in Oncology and Neurology, Department of Pulmonary Oncology, The First People’s Hospital of Foshan, Foshan, Guangdong 528000 China; 5 School of Marine Sciences, Sun Yat-sen University, Zhuhai, Guangdong 519082 China; 6 Guangdong Engineering Technology Research Center of Urinary Continence and Reproductive Medicine, The Affiliated Qingyuan Hospital (Qingyuan People’s Hospital), Guangzhou Medical University, Qingyuan, Guangdong 511518 China; 7 Department of Radiation Oncology, Zhujiang Hospital, Southern Medical University, Guangzhou, Guangdong 510280 China; 8 Guangdong Provincial Key Laboratory of New Drug Design and Evaluation, State Key Laboratory of Anti-Infective Drug Discovery and Development, Sun Yat-Sen University, Guangzhou, Guangdong 510006 China; 9 Guangdong Lung Cancer Institute, Guangdong Provincial People’s Hospital, Guangdong Academy of Medical Sciences, Southern Medical University, Guangzhou, Guangdong 510080 China

**Keywords:** Non-small cell lung cancer, RORγ, Antagonist, NGF, Therapeutic target

## Abstract

**Background:**

Lung cancer remains one of the most prevalent and lethal malignancies worldwide, with non-small cell lung cancer (NSCLC) representing the most common subtype—highlighting the critical need for novel therapeutic approaches. The retinoic acid receptor-related orphan receptor gamma (RORγ) has been implicated in various cancers, but its role and mechanism in NSCLC remain unclear.

**Methods:**

RORγ expression and its correlation with patient prognosis in NSCLC were assessed by integrating public database bioinformatics analysis, immunohistochemistry, and Western blot. The functional roles of RORγ in NSCLC proliferation, migration, and invasion were determined in vitro through genetic overexpression, pharmacological inhibition, or genetic silencing of RORγ, assessed by cell counting, colony formation, wound healing, and transwell assays. The underlying mechanism was investigated using RNA sequencing, chromatin immunoprecipitation, and rescue experiments with exogenous nerve growth factor (NGF) supplementation or NGF overexpression. In vivo, the anti-tumor efficacy of RORγ inhibition was evaluated using subcutaneous xenograft and experimental metastasis models.

**Results:**

We identify RORγ as a key driver of NSCLC progression. Integrative bioinformatics and immunohistochemical analysis revealed that RORγ is highly expressed in NSCLC tissues and that its expression correlates with poor patient prognosis. Functionally, elevated RORγ significantly enhanced the proliferation, migration, and invasion capabilities of NSCLC cells. Conversely, treatment with the RORγ antagonist or genetic silencing of RORγ potently suppressed these malignant phenotypes both in vitro and in vivo. Mechanistically, RORγ directly binds to the promoter region of NGF, stimulates NGF gene transcription, and thereby promotes NSCLC progression. RORγ antagonists suppress NGF expression and inhibit its downstream signaling pathways, whereas exogenous NGF supplementation or overexpression of NGF notably reverses the inhibitory effects of RORγ antagonists on NSCLC cells.

**Conclusion:**

Taken together, these results establish RORγ as a critical regulator of NSCLC and a promising therapeutic target for NSCLC treatment.

**Supplementary Information:**

The online version contains supplementary material available at 10.1186/s12931-026-03523-7.

## Introduction

Lung cancer is a malignant tumor with the highest global incidence and mortality rates [1]. Non-small cell lung cancer (NSCLC), the most prevalent form of the disease, accounts for approximately 85% of all lung cancer cases [2]. Recent breakthroughs in targeted therapies have significantly improved the clinical management of NSCLC and enhanced the prognosis for many patients. For those with sensitive driver gene mutations, such as EGFR mutations or ALK fusion genes, targeted therapy has become the first-line treatment option for NSCLC, significantly prolongs progression-free survival (PFS) [3]. However, despite these advances, tumor heterogeneity and acquired drug resistance frequently led to disease recurrence and metastasis, which continue to contribute to poor long-term outcomes. Therefore, a deeper understanding of the molecular mechanisms driving NSCLC progression is urgently needed to identify novel therapeutic targets and develop more effective treatment strategies.

RAR-related orphan receptor gamma (RORγ), encoded by RORC, along with RORα and RORβ, constitutes a subfamily of nuclear receptors that display distinct tissue expression patterns and likely serve different physiological functions [4–7]. The development of synthetic ligands targeting these receptors is currently in progress for potential therapeutic uses in immune-related disorders, metabolic conditions, circadian rhythm disruptions, and cancer [7]. RORγ has two isoforms, commonly known as RORγ and RORγt. RORγt is specifically expressed in certain immune cells, including T helper 17 cells (Th17), γδT cells, and innate lymphoid cells like ILC3, through an immune cell-specific promoter [8–10]. Given RORγt’s crucial role in inflammatory and autoimmune diseases, it is regarded as a promising target for therapeutic strategies in treating these conditions [11]. Recent studies, including our own, have shown that RORγ plays a significant role in the tumor growth and metastasis of prostate cancer, hepatocellular carcinoma, and pancreatic cancer by regulating critical pathways such as androgen receptor signaling, extracellular matrix remodeling, and the behavior of cancer stem-like cells [4, 6, 12]. However, the role of RORγ in non-small cell lung cancer (NSCLC) remains unclear and warrants further exploration.

In this study, we aimed to investigate the function of RORγ in non-small cell lung cancer (NSCLC) and evaluate its potential as a therapeutic target to develop novel treatment strategies for this lethal disease. We found that RORγ is highly expressed in NSCLC and that its expression is correlated with poorer prognosis. Using gain and loss of function studies, we identify RORγ as a critical driver of NSCLC tumor progression. Mechanistic studies demonstrated that RORγ promotes NSCLC malignancy by directly stimulating NGF gene transcription and activating its downstream signaling pathway. Thus, our findings establish RORγ as a promising therapeutic target for NSCLC treatment.

## Materials and methods

### Cell lines and cell culture

The HEK293T cells and human NSCLC cell line A549 were obtained from the American Type Culture Collection (ATCC). Human NSCLC cell line H157 was obtained by Procell Life Science & Technology (Wuhan, China), while the murine LLC1 cell line was purchased from the China Academia Sinica (Shanghai, China). The HEK293T cells and LLC1 cells were cultured in high-glucose DMEM completed medium, containing 10% fetal bovine serum (FBS, Excell) and 1% penicillin/streptomycin (Gibco), while A549 and H157 cells were maintained in RPMI-1640 completed medium. All cells were incubated at 37 °C with 5% CO₂ in a humidified atmosphere.

### Patient specimens

The normal and tumor tissues from patients were obtained from The Third Affiliated Hospital of Sun Yat-sen University, and all specimens were histologically confirmed by biopsy. The clinical research protocols involving human samples were approved by the Clinical Ethics Committee of The Third Affiliated Hospital of Sun Yat-sen University (Approved number: RG2024-080-01).

### Reagents and antibodies

The RORγ antagonists XY018 and W6134 were synthesized, purified, and structurally characterized by the group of Wang Yuanxiang, as described previously [12, 13]. The primary antibodies against NGF, ERK and phosphorylated ERK (p-ERK), were purchased from Santa Cruz Biotechnology (Dallas, USA). RORγ antibody was purchased from Proteintech (Wuhan, China). GAPDH antibody, anti-rabbit IgG Fab2, and anti-mouse IgG Fab2 were obtained from Cell Signaling Technology (Danvers, USA).

### Cell growth and colony formation assay

To evaluate cell growth, A549 and H157 cells were plated in 6-well plates at a density of 4 × 10^4^ cells per well and incubated with RORγ antagonist W6134 for 96 h. Cell counts were determined by Coulter counter analysis.

To evaluate the effects of RORγ antagonists on cell growth, A549 and H157 cells were seeded in 6-well plates at a density of 800 cells per well and treated with RORγ antagonists for 7–12 days. Besides, A549 and H157 cells (wide type and RORC overexpression type) were seeded in 6-well plates at a same density to investigate the functional role of RORγ in NSCLC cell proliferation. The medium was changed every 3 days and added RORγ antagonists at indicated concentrations. Upon colony formation, the medium was removed, and fresh PBS was used to wash colonies for 3 times. Colonies then fixed in 4% paraformaldehyde for 15 min, and washed in the same method, and subsequently stained with 0.1% crystal violet solution under light-protected condition for 30 min. After washing the cells with PBS, colonies were air-dried and photographed under bright-field microscopy.

### Western blotting analysis

Protein lysates from NSCLC cells or paired normal and tumor tissues of patients were extracted using RIPA lysis buffer (Beyotime, Shanghai, China) containing protease inhibitors (Beyotime, Shanghai, China) and phosphatase inhibitors (Bimake, USA). Protein concentrations were determined by BCA assay (Thermo, USA) with bovine serum albumin as the standard. Equal amounts of proteins were loaded on 8%-15% SDS-page gels and separated with 80–120 V for 90–120 min according to the molecular weight of the target proteins. Subsequently, the proteins on the gels were electrophoretically transferred onto PVDF membrane using a wet transfer system under ice-cooled conditions at 235 mA constant current mode for 2 h. Then, post-transfer membranes were blocked with 5% non-fat milk in TBST for 1 h at room temperature, followed by overnight incubation at 4 ℃ with primary antibodies diluted in blocking buffer. The next day, the protein bands were incubated at room temperature with HRP-conjugated secondary antibodies for 1 h. After TBST washing 3 times, appropriate volume ECL was added onto the surface of membranes to incubate several minutes before visualization using a ChemiDoc Molecular Imager system.

### SiRNA transfection and lentivirus infection

siRNAs targeting RORC and NGF genes were purchased from Sangon Biotech (Shanghai, China). The siRNA sequences of RORC and NGF were supplied on Supplementary Table S1. NSCLC cells were seeded in 6-well plates at a suitable density for the overnight incubation and transfected with siRNA using Opti-MEM and DharmaFECT (Dharmacon, USA) following the manufacturer’s guidelines. Lentiviral particles packaged in HEK293T cells were transfected into A549 and H157 cells. For the selection of blasticidin-resistant clones, the culture medium was supplemented with 5 µg/mL blasticidin for a duration of 2 weeks. Then single-cell sorting was performed in 96-well plates to expand single clones that were verified for the overexpression efficiency by Western blotting analysis.

### Migration and invasion assays

NSCLC cells were seeded in 6-well plates at a suitable density and cultured overnight until they grow to reach 90% confluency. A sterile pipette tip was used to generate a linear wound in the cell monolayer, followed by microscopic imaging steps. Removed debris with 2 times PBS washing, and fresh complete medium containing W6134 at gradient concentrations was added. Captured 3 images for each concentration. Wound closure was assessed after 24 h by comparing the remaining gap area to the initial measurement, with healing percentage calculated using ImageJ.

For invasion evaluation, 24-wells Matrigel-coated Transwell^®^ chambers were used. Before adding cells, serum-free medium was added into the transwell chambers until complete submersion was achieved, and incubated for 1 h. Following trypsinization, 2 × 10^4^cells in 300 µL serum-free medium were loaded into each upper chamber, while the lower compartment contained 500 µL complete medium as chemoattractant. After 24 h incubation, migrated cells on the membrane underside were fixed with 4% PFA for 15 min, and stained with 0.1% crystal violet for 30 min. Three random fields were imaged under the microscope and Image J were used to count the cells for statistical analysis.

### Total RNA extraction and RNA-seq analysis

A549 and H157 cells were treated with vehicle (DMSO) or W6134 (2.5 µM) for 48 h before total RNA extraction. The extraction steps of total RNA were according to the manufacturer’s protocol of the Vazyme Super FastPure Cell RNA Isolation Kit (Vazyme Biotech, Nanjing, China). Transcriptomic sequencing of RNA-seq libraries were validated through MGISEQ2000 machine (BGI Tech, China). Enrichment analysis of co-downregulated genes (expression changes of ≥ 1.5-fold) in both cell lines following W6134 treatment was performed using R (v4.2.1), with the most significantly enriched pathways visualized via volcano plots (considering *p* adjust (FDR) < 0.05 as statistically significant).

### Quantitative real-time PCR and ChIP-qPCR

Super FastPure Cell RNA Isolation Kit was used to extract the total cellular RNA. Following genomic DNA removal, total RNA was reverse transcribed into cDNA. According to the manufacturer’s instructions. The cDNA was amplified by combining SYBR Green master mix (YEASEN, Shanghai, China) and gene-specific primers, then BIO-RAD CFX96 (Bio-Rad) was used to detect and finish the real-time PCR. Following fluorescence data acquisition, melting curve analysis was conducted to verify amplicon specificity. The corresponding primer sequences are detailed in Supplementary Table S2.

The ChIP-qPCR assay was performed as our previous work [12]. Briefly, A549 cells, pre-treated with 2.5 µM W6134 for 48 h, were subjected to protein-DNA crosslinking with 1% paraformaldehyde, followed by glycine quenching. Nuclear extracts were prepared by cell lysis after collection and centrifugation. Chromatin was sheared from the isolated nuclei using a Covaris E220 instrument. Specific protein-DNA complexes were immunoprecipitated from the sheared chromatin using antibodies coupled to Protein G beads. After reverse cross-linking and DNA purification, the recovered DNA was analyzed via real-time PCR. The ChIP-qPCR primer sequences are provided in Supplementary Table S3.

### Reporter-gene assays

For RORE reporter assays, HEK293T cells were transfected with the NGF-RORE-luciferase reporter plasmid, a RORγ expression plasmid (in pLX304), and a renilla luciferase plasmid as an internal control. The NGF-RORE mutant contains sequences mutated from CTGGGGCA to CTGAACGA. After 24 h, cells were treated with vehicle or W6134 (2.5 µM) for an additional 24 h. Luciferase activity was then measured using the Dual-Luciferase Assay system (Promega) on a luminometer according to the manufacturer’s protocol.

### Cell rescue assay

High-viability A549 and H157 cells were seeded in 6-well plates at a density of 1 × 10^5^ cells per well. After 24 h of adherence, cells were treated with either exogenous NGF (100 ng/mL) or transfected NGF-overexpressing viral particles, followed by a 24-hour incubation. For viral-transfection groups, the culture medium was replaced with fresh complete medium at the 16-hour time point. Following NGF treatment, cells were grouped. The treatment groups were exposed to W6134 at the indicated concentrations, while control groups were given an equal volume of DMSO. All groups were cultured for an additional 24 h to assess drug effects. Finally, cells were harvested, counted, and analyzed using statistical software.

### Immunohistochemistry (IHC)

5 paired normal and tumor specimens from NSCLC patients were fixed in 10% neutral buffered formalin for 24 h and paraffin-embedded. Paraffin-embedded tissue blocks underwent sequential dewaxing in xylene, rehydration through graded alcohols, and endogenous peroxidase blockade with 3% H₂O₂. Following antigen retrieval, 5-µm sections were probed with primary antibodies overnight in a humidified chamber at 4℃ cool condition. After 3 times PBS washes, HRP-conjugated secondary antibodies were applied to combine the primary antibodies at room temperature for 1 h. DAB chromogenic development (Servicebio, Wuhan, China) and hematoxylin counter staining preceded final dehydration and neutral resin mounting. Digital images were acquired using a microscope with imaging software.

### Animal experiments

The experimental protocols involving animals were approved by the Institutional Animal Care and Use Committee (IACUC) of Sun Yat-sen University (Approval numbers: SYSU-IACUC-2024-003186 and 000067), ensuring full compliance with national animal welfare regulations and the 3Rs principles. Animals used in these experiments including male C57BL/6 and BALB/c-nu/nu mice (aged 4–6 weeks, weighing 16–20 g) were purchased from the Laboratory Animal Center of Sun Yat-sen University. All animals were maintained under specific pathogen-free (SPF) conditions.

CDX (cell line-derived xenograft) models for LLC1: High-viability murine NSCLC LLC1 cells were resuspended in a cell suspension with a PBS: Matrigel matrix ratio of 3:1, where each 100µL of the suspension contained 2.5 × 10^5^ cells. The cells were then implanted at 100µL per side into the dorsal subcutaneous region of each C57BL/6 mouse thigh. When the average tumor volume reached 100 mm³, mice were randomly divided into XY018 (10 mg/kg) group, XY018 (20 mg/kg) group, W6134 (20 mg/kg) group, and control group, with 8 mice per group. Mice were administered daily with RORγ antagonists or physiological saline, and body weight and tumor volume were measured every 2–3 days. The tumor volume was calculated using the formula: Π/(length × width^2^). At the end of the experiment, mice were sacrificed, and tumor weight was measured. Major organs were immersed in tissue fixative and subjected to H&E staining.

CDX models for A549: High-viability A549 cells were transinfected with shRORC lentivirus and continually cultured for 24 h. The remaining experimental procedures followed the same steps as the CDX models for LLC1.

To evaluate effects on lung colonization, C57BL/6 mice were injected via the tail vein with 2 × 10^5^ luciferase-expressing LLC1 cells per mouse. The mice were then treated daily via intraperitoneal injection with either vehicle, 10 mg/kg, or 20 mg/kg of W6134 for 4 weeks. Lung tumor growth was monitored weekly using bioluminescence imaging with a PerkinElmer IVIS Spectrum system. For histological analysis, mice were euthanized at the end of the treatment period, and lung tumor nodules were examined using H&E staining for morphological assessment.

### Bioinformation analysis

The analysis of RORs mRNA levels in LUAD (lung adenocarcinoma) tumors was from the public database cBioPortal for Cancer Genomics. The single-cell RNA seq-data from primary tumor tissues of 58 NSCLC patients were used to analyze the expression levels of RORγ in different cell types within tumor tissues. The single-cell data were downloaded from the NCBI-GEO database (GSE131907). Rstudio 4.3 software with the edgeR package was used to analyze the single-cell transcriptome data from patients. Cell types were annotated using the original metadata file (GSE131907_Lung_Cancer_cell_annotation.txt.gz) provided with the dataset, which was generated based on established marker genes for major lung and immune cell lineages. *RORC* expression in epithelial cells was defined by comparative analysis across all annotated cell types. Survival curves for NSCLC patients were analyzed using the online Kaplan–Meier plotter (KMplotter) tool.

### Data and code availability

All associated data supporting this study are available upon request from the corresponding authors. Gene expression data from RNA-seq analysis were deposited in the NCBI GEO database (GSE308540).

### Statistical analysis

GraphPad Prism 8.0 software (GraphPad Software, USA) was used for statistical analysis and plotting of the experimental data in this chapter. Statistical results of in vitro experiments were presented as mean ± SD, while tumor volume changes in animal experiments were presented as mean ± SEM. Intergroup differences in each experiment were statistically analyzed using the Student’s *t*-test. *p < 0.05 was considered significant.

## Results

### RORγ is overexpressed in NSCLC tumors and predicts poor prognosis

**Fig. 1:**
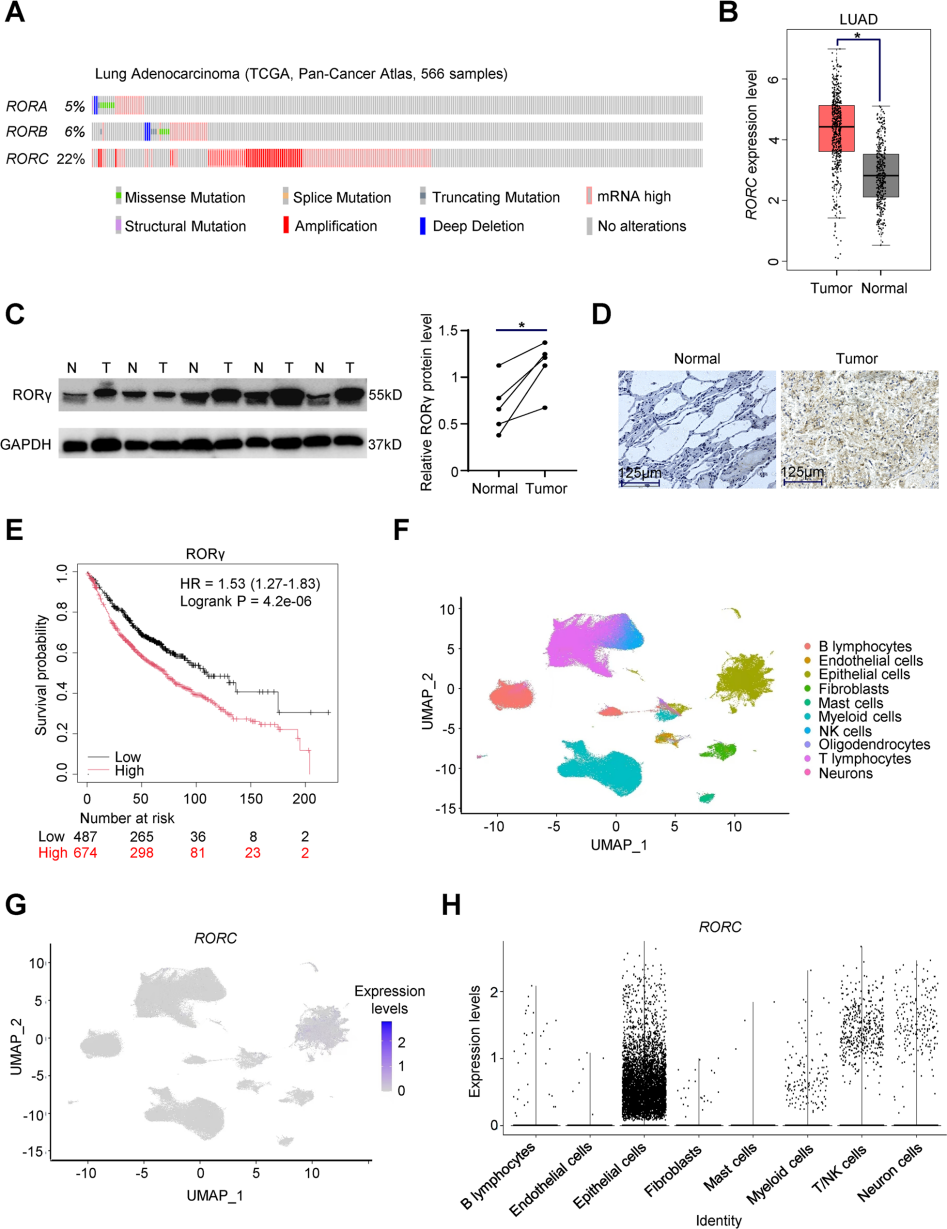
RORγ is overexpressed in NSCLC tumors and predicts poor prognosis.** (A)** cBioPortal platform was used to obtain mRNA amplification profile of RORs family in NSCLC patients. **(B)** In the TCGA database, the RORγ protein expression level of LUAD patients was higher in NSCLC tumors (*n* = 483) than in normal tissues (*n* = 347). **(C)** Western blotting was used to detected the RORγ protein level in 5 paired NSCLC tumors (T) and adjacent normal tissues (N), and the result of western blot was quantified by ImageJ software. **(D)** Immunohistochemistry staining was used to detected the protein level of RORγ expressed in NSCLC tissues and normal tissues (*n* = 5). Scale bars, 125 μm. **(E)** The survival curve of the relationship between RORγ expression and NSCLC patient’s prognosis was drawn via KM-plotter (Low = 487, High = 674). **(F)** Based genomic characteristics, the single cells were grouped into distinct clusters. *n* = 58. **(G)** The mRNA expression level of RORγ in distinct clusters from (F). **(H)** The expression level of RORγ in epithelial cells was higher than other cell types. The mean ± SD was used to show the results of all data. *p < 0.05. Unpaired Student’s *t* test (two-tailed) is used in (**B**). Paired Student’s *t* test (two-tailed) is used in (**C**). Log-rank test is used in (**E**)

To investigate the potential role of the nuclear receptor RORγ in NSCLC malignant progression, we analyzed gene copy number variation data from tumor samples of 566 LUAD (lung adenocarcinoma) patients in the TCGA database. This analysis revealed significant RORγ gene amplification in LUAD patients (Fig. 1A). Further analysis of RORγ protein expression levels in normal and tumor tissues from LUAD patients within the TCGA database showed a significantly higher expression level of RORγ in tumor tissues compared to normal lung tissues (Fig. 1B). To validate these bioinformatic findings, we assessed RORγ expression levels via Western blotting and IHC in tumor tissues and paired adjacent non-tumor tissues from NSCLC patients. Consistent with the TCGA data, RORγ expression was significantly elevated in tumor tissues (Fig. 1C and D). Using the KM-plotter database, we analyzed survival curves of 1,161 NSCLC patients and found that high RORγ expression was significantly associated with poorer prognosis (Fig. 1E). To identify the specific cell types within NSCLC tumors exhibiting high RORγ expression, we analyzed single-cell RNA sequencing data from NSCLC patient samples. Dimensionality reduction and clustering analysis classified single-cell data from 58 NSCLC patients into 10 distinct cell subpopulations (Fig. 1F). Enrichment analysis indicated that RORγ was primarily expressed in epithelial cells, with relatively high expression also detected in lymphocytes and T/NK cells (Fig. 1G and H). These findings suggest that elevated RORγ expression in epithelial cells may be a key driver of NSCLC progression.

### RORγ overexpression drives NSCLC tumor cell growth and invasion

**Fig. 2:**
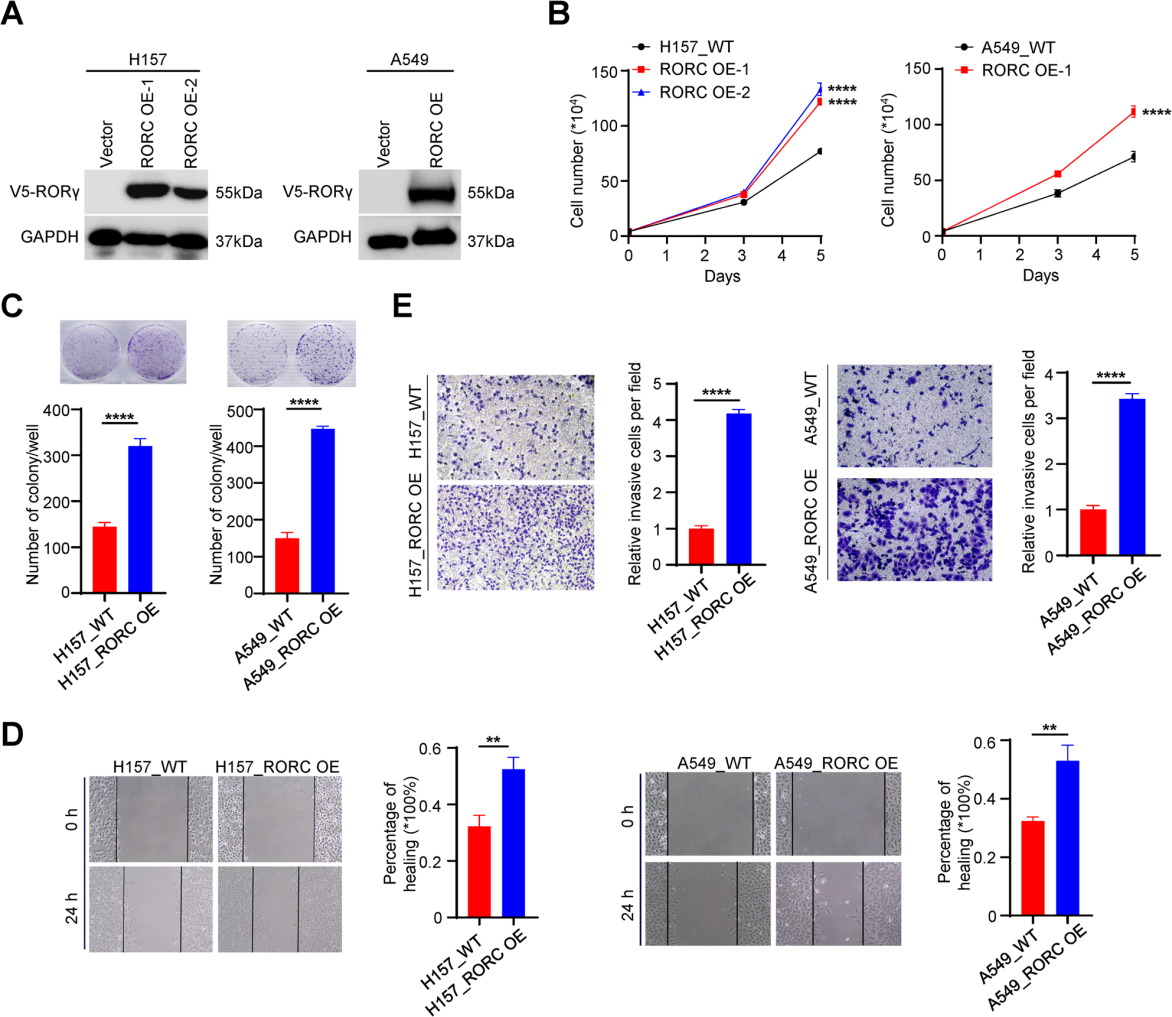
RORγ overexpression drives NSCLC tumor cell growth and invasion.** (A)** Validate the protein level of RORγ after over-expression in NSCLC cells by western blotting. **(B)** NSCLC cells or colonies **(C)** of the RORC overexpression were counted at certain time points. *n* = 3. **(D)** The migration of NSCLC cells with/without RORC overexpression. *n* = 3. **(E)** The invasion of NSCLC cells with/without RORC overexpression. *n* = 3. The mean ± SD was used to show the results of all data. **p < 0.01, ****p < 0.0001. Unpaired Student’s *t* test (two-tailed) is used in (**A-E**)

To examine the function of RORγ in NSCLC cells, we first generated RORγ-overexpressing sublines in the A549 and H157 cell lines using lentiviral transduction (Fig. 2A). The results showed that elevated RORγ expression alone was sufficient to stimulate NSCLC cell growth (Fig. 2B). A colony formation assay further demonstrated that RORγ overexpression significantly enhanced NSCLC cell survival (Fig. 2C). Given that metastasis remains a major challenge in NSCLC treatment, we next investigated whether RORγ regulates the migration and invasion of NSCLC cells. Indeed, elevated RORγ expression significantly promoted the migratory and invasive capabilities of A549 and H157 cells, as measured by scratch wound healing and transwell assays (Fig. 2D and E). These findings suggest that RORγ overexpression drives the growth, migration, and invasion of NSCLC cells.

### RORγ inhibition suppress the growth and invasion of NSCLC cells

**Fig. 3:**
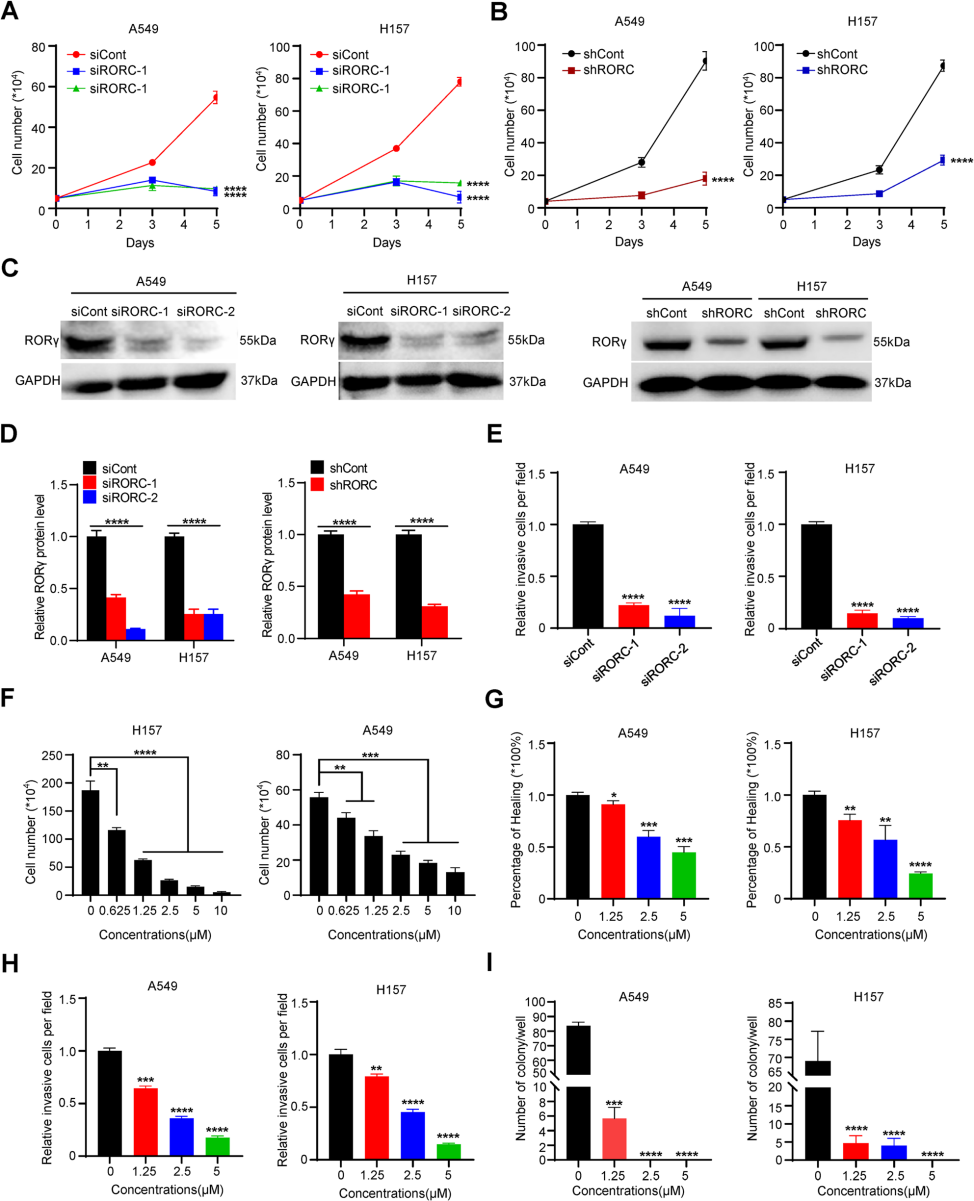
RORγ inhibition suppress the growth and invasion of NSCLC cells. **(A**,** B)** Cell numbers of NSCLC cells infected with RORC siRNA or shRNA were counted at certain time points. *n* = 3. **(C**,** D)** Validate the protein level of RORγ after knocking down in NSCLC cells by western blotting. Representative blots are shown, accompanied by densitometric quantification (bar graph). **(E)** The invasion assay of NSCLC cells infected with Negative control or RORC siRNA. *n* = 3. **(F)** Cell numbers of NSCLC cells treated with RORγ antagonist were counted at certain time points. *n* = 3. **(G)** The migration assay of NSCLC cells treated with DMSO/W6134. *n* = 3. **(H)** The invasion assay of NSCLC cells treated with DMSO/W6134. *n* = 3. **(I)** Colonies of NSCLC cells treated with RORγ antagonist. *n* = 3. The mean ± SD was used to show the results of all data. *p < 0.05, **p < 0.01, ***p < 0.001, ****p < 0.0001. Unpaired Student’s *t* test (two-tailed) is used in (**A**,** B**,** D-I**)

To further validate the functional significance of RORγ in NSCLC cell survival, we employed siRNA and shRNA to specifically silence RORγ expression in A549 and H157 cell lines. As shown in Fig. 3A-D, RORγ knockdown significantly suppressed NSCLC cell growth. Furthermore, RORγ depletion markedly reduced the invasive capacity of NSCLC cells in vitro (Fig. 3E). To corroborate these findings, we evaluated the effects of W6134, a potent covalent RORγ antagonist, on NSCLC cell behavior. Consistent with the results from RORγ knockdown experiments, W6134 treatment robustly inhibited cell growth, migration, and invasion in NSCLC cells (Fig. 3F-H). Notably, W6134 also induced a significant reduction in clonogenic survival, as demonstrated by colony formation assays (Fig. 3I). Collectively, these findings establish RORγ as a critical regulator of NSCLC cell survival and invasiveness.

### RORγ directly controls*NGF*gene expression

**Fig. 4:**
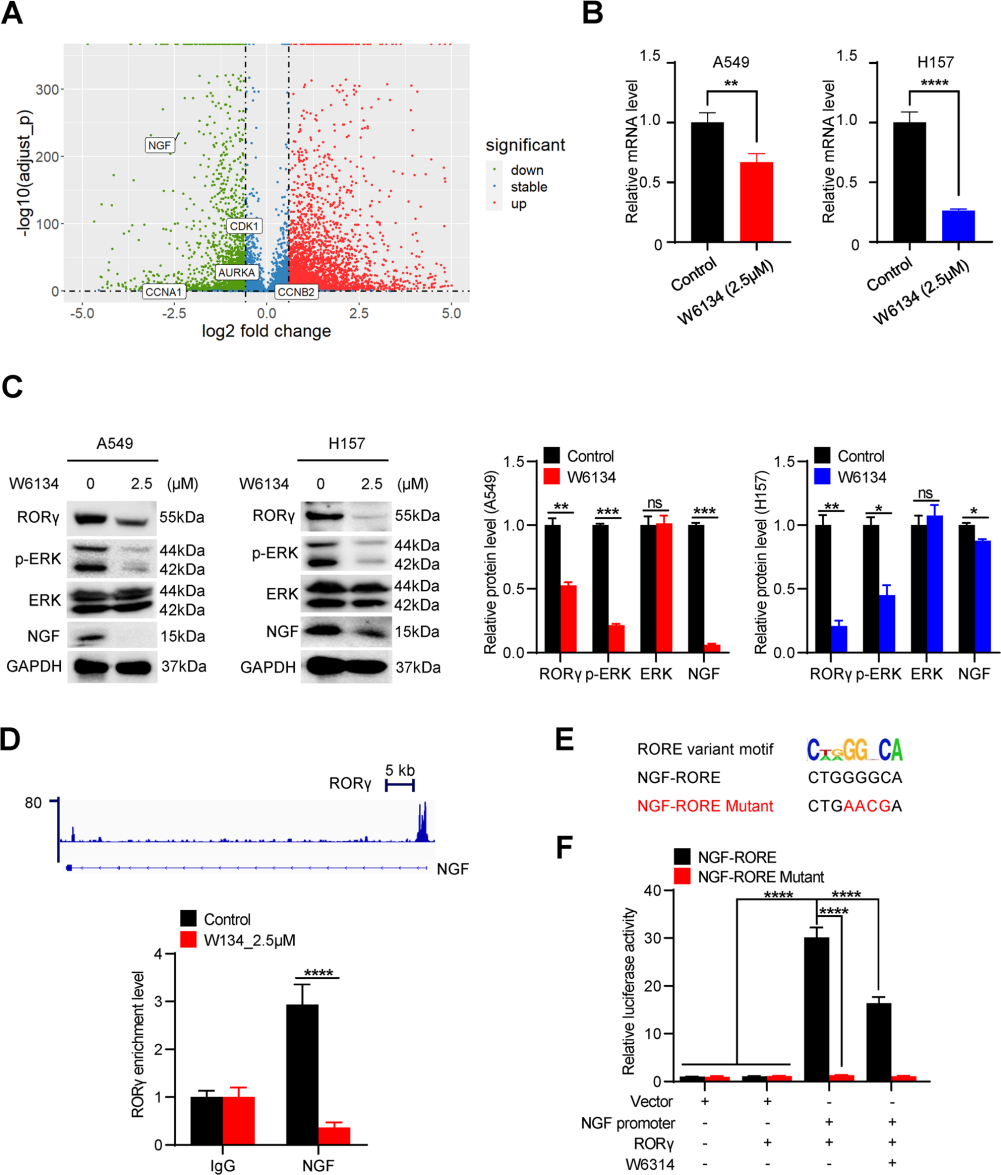
RORγ directly controls *NGF* gene expression.** (A)** Volcano plot reflecting gene expression alterations after W6134 (2.5µM) treatment for 48 h. **(B)** The mRNA expression level of NGF in A549 and H157 cells treated by W6134 for 48 h was determined by qRT-PCR. *n* = 3. **(C)** The protein level of NGF and downstream makers with W6134 treatment in A549 and H157 cells was detected by western blotting. Representative blots are shown, accompanied by densitometric quantification (bar graph). **(D)** ChIP-qPCR was used to confirm the combination of RORγ on the NGF promoter region in A549 cells. *n* = 3. **(E)** Sequences of the wild type and mutant (in red) forms of NGF-RORE. **(F)** NGF-RORE (wild type or RORE mutated) reporter-gene activity changes in 293T cells co-transfected with RORC for 24 h, and then treated with W6134 (2.5µM) for another 24 h. *n* = 5. All data shown as the mean ± SD. *p < 0.05, **p < 0.01, ***p < 0.001, ****p < 0.0001. ‘‘ns’’ means no significant. Unpaired Student’s *t* test (two-tailed) is used in (**B-D**,** F**)

To elucidate the molecular mechanism by which RORγ regulates NSCLC cell survival and invasion, we performed transcriptomic profiling using RNA sequencing (RNA-seq) in A549 and H157 cells treated with the RORγ antagonist W6134. Differentially expressed genes (DEGs) showing consistent upregulation or downregulation (≥ 1.5-fold change) in both cell lines were selected for further analysis. As shown in Fig. 4A, nerve growth factor (NGF) was identified as one of the most significantly downregulated genes following W6134 treatment. Emerging evidence has demonstrated that NGF plays a critical role in tumor growth and metastasis across multiple cancer types. Therefore, we hypothesized that RORγ may modulate NSCLC progression through regulation of the NGF signaling pathway.

To validate the RNA-seq results, we conducted qRT-PCR and immunoblotting assays in NSCLC cells treated with W6134. Consistent with the RNA-seq data, W6134 significantly reduced NGF expression at both the mRNA and protein levels (Fig. 4B and C). Moreover, W6134 treatment markedly inhibited the phosphorylation of ERK, a key downstream effector of NGF signaling (Fig. 4C). To determine whether RORγ directly regulates NGF transcription, we analyzed our previously published RORγ chromatin immunoprecipitation sequencing (ChIP-seq) data from cancer cells. This analysis revealed that RORγ binds to the promoter region of the NGF gene (Fig. 4D). Subsequent ChIP-qPCR experiments confirmed RORγ occupancy at the NGF promoter locus, and this binding was significantly reduced in W6134-treated cells (Fig. 4D). Previous studies demonstrated that RORγ binds DNA with the specific response elements A(A/T)NTAGGTCA (the classic RORE motif) or C(T/A)(G/A) GGNCA (the variant RORE motif) [12, 14]. We then performed reporter gene assays on the RORE within the NGF gene and found that it was highly responsive to RORγ–mediated transactivation. Mutation in the core RORE sequence completely abrogated the NGF-RORE–dependent activation. Importantly, the RORγ antagonist W6134 suppressed this activation (Fig. 4E and F). Collectively, these findings demonstrate that RORγ directly activates NGF transcription in NSCLC cells.

### RORγ drives NSCLC cell survival and invasion through upregulation of NGF signaling

**Fig. 5:**
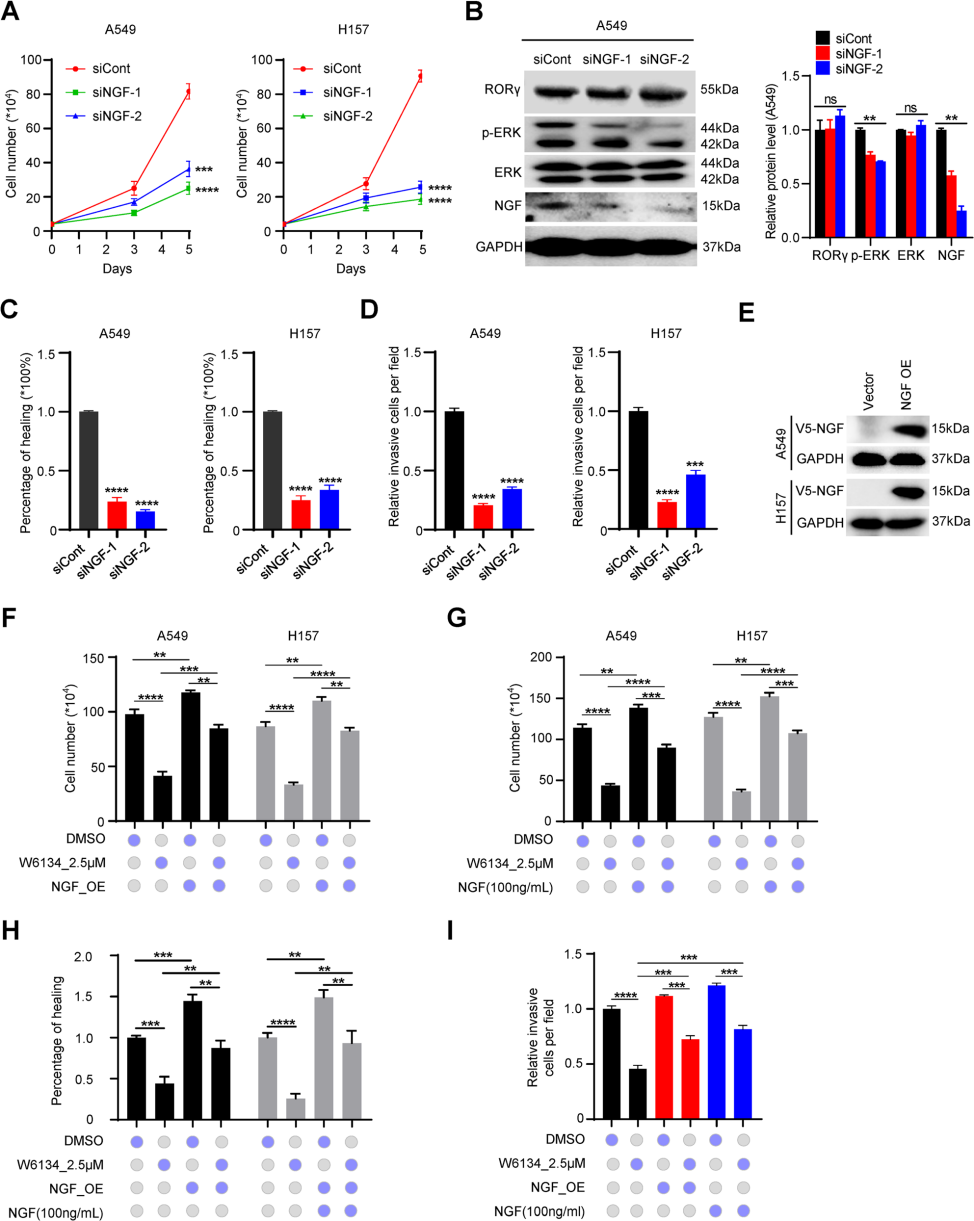
RORγ drives NSCLC cell survival and invasion through upregulation of NGF signaling.** (A)** NSCLC cells were infected with NGF siRNA. Three and five days later, viable cell numbers were counted. *n* = 3. **(B)** Validate the protein level of NGF and its associated downstream makers by Western blotting after NSCLC cells infected with NGF siRNA. Representative blots are shown, accompanied by densitometric quantification (bar graph). **(C**,** D)** The migration **(C)** and invasion **(D)** assay of NSCLC cells infected with NGF siRNA. *n* = 3. **(E)** Validate the protein level of NGF after over-expression in NSCLC cells by western blotting. Representative blots are shown. **(F**,** G)** NGF is over-expressed or NGF recombinant protein added into A549 and H157 cells, viable cell numbers were counted after W6134 treatment. *n* = 3. **(H**,** I)** Wound healing assay **(H)** and transwell invasion assay **(I)** were quantitative analysis. *n* = 3. All data shown as the mean ± SD. **p < 0.01, ***p < 0.001, ****p < 0.0001. ‘‘ns’’ means no significant. Unpaired Student’s *t* test (two-tailed) is used in (**A -I**)

Given our findings that RORγ promotes cell survival, invasion and upregulates FGF1 expression in NSCLC cells, we investigated whether RORγ drives NSCLC cell survival and invasion through activation of NGF signaling. First, to determine the role of NGF in NSCLC cells, we observed that consistent with RORγ inhibition, NGF knockdown significantly reduced ERK phosphorylation and markedly suppressed NSCLC cell proliferation, migration, and invasion (Fig. 5A-D). Next, to test whether RORγ exerts its regulatory effects on NSCLC progression via NGF signaling, we performed rescue experiments by either overexpressing NGF or supplementing exogenous NGF in NSCLC cells. The results revealed that both NGF overexpression and exogenous NGF treatment effectively reversed the inhibitory effects of the RORγ antagonist W6134 on cell proliferation, migration, and invasion (Fig. 5E-I). Collectively, these data demonstrate that RORγ promotes NSCLC cell survival and invasion through activation of NGF signaling.

### Targeting RORγ inhibits NSCLC tumor progression *in vivo*

**Fig. 6:**
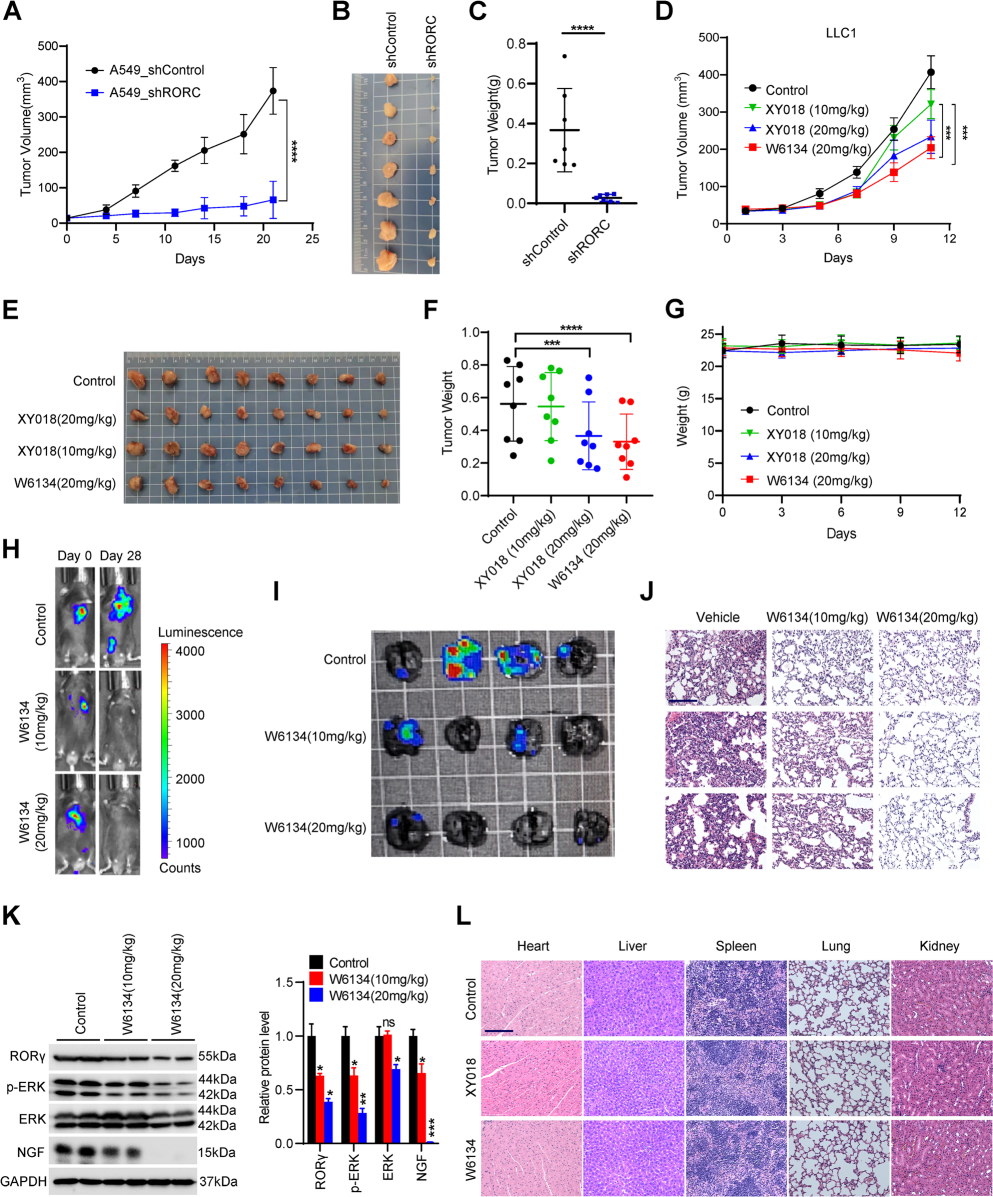
Targeting RORγ inhibits NSCLC tumor progression *in vivo.* (**A-C)** Male Balb/c-nude mice bearing A549 or A549 shRORC subcutaneous xenografts. **(A)** The volume of subcutaneous tumor in vivo was measured every 2 or 3 days (*n* = 7). **(B)** Representative images of the tumor from mice were captured. **(C)** Tumor weight was measured. (**D-G)** Male C57BL/6 mice bearing LLC1 subcutaneous xenografts received W6134 (i.p., 20 mg/kg), XY018 (i.p., 10 mg/kg and 20 mg/kg) or vehicle per day for 12 days. **(D)** The volume of subcutaneous tumor in vivo was measured every 2 or 3 days (*n* = 8). **(E)** Representative images of the tumor from mice were captured. **(F)** Tumor weight was measured on the last day. **(G)** The body weight was measured every 3 days. (**H-J)** LLC1 cells with EGFP luciferase were intravenously injected into the tail veins of C57BL/6 mice to establish pulmonary metastasis models (*n* = 4). **(H)** Bioluminescent images of in vivo tumor growth status for the first and last day. **(I)** Bioluminescent images of ex vivo tumor tissues for the last day. **(J)** H༆E staining was used to evaluate the inhibition effects of W6134 on the pulmonary metastasis. Scale bars, 125 μm. **(K)** The protein expression level of RORγ, NGF and its associated downstream makers was detected by western blotting. Representative blots are shown, accompanied by densitometric quantification (bar graph). **(L)** H༆E staining was used to evaluate the toxic effects of W6134 on the major organs of mice with tumors. Scale bars, 125 μm. The mean ± SEM was used to show the results of tumor volume. *p < 0.05, **p < 0.01, ***p < 0.001, ****p < 0.0001. ‘‘ns’’ means no significant. Unpaired Student’s *t* test (two-tailed) is used in (**A**,** C**,** D**,** F**,** K**)

Given the pronounced suppression of cell survival by RORγ inhibition, we further evaluated the antitumor efficacy of targeting RORγ in NSCLC. Consistent with in vitro findings, RORγ knockdown significantly inhibited tumor formation and growth in A549 xenograft models (Fig. 6A-C). To assess therapeutic effects in an immune-competent context, we tested RORγ antagonists in LLC1 syngeneic tumor models. Notably, the RORγ antagonist W6134 markedly suppressed LLC1 tumor growth in immune-intact hosts (Fig. 6D-G). To investigate antimetastatic potential, we established LLC1 lung metastasis models via tail vein injection and administered W6134. Bioluminescence imaging revealed a significant reduction in metastatic burden in W6134-treated mice (Fig. 6H and I). Histopathological analysis further confirmed diminished pulmonary metastasis in treated cohorts (Fig. 6J). Mechanistically, residual tumors from W6134-treated groups exhibited reduced NGF expression (Fig. 6K), aligning with our earlier mechanistic insights. Notably, RORγ antagonists were well tolerated, with no adverse effects on body weight, organ histopathology, or behavioral parameters (e.g., food/water intake, grooming, and activity) (Fig. 6G and L). Collectively, these findings validate RORγ as a therapeutically actionable target in NSCLC, offering a dual benefit of inhibiting both tumor growth and metastasis.

## Discussion

Lung cancer remains the leading cause of cancer-related mortality due to limited therapeutic options. Here, we demonstrate that RORγ represents a promising therapeutic target for non-small cell lung cancer (NSCLC). Our analysis revealed significantly elevated RORγ expression in NSCLC tumors, with high RORγ levels correlating with poor patient prognosis. Genetic gain- and loss-of-function studies established that RORγ enhances NSCLC cell growth and invasion by directly activating NGF signaling. Pharmacological inhibition of RORγ with a selective antagonist or genetic RORγ silencing potently suppressed tumor growth and metastasis in vivo. Given the favorable druggability of RORγ, our findings position RORγ as a highly actionable therapeutic target for NSCLC, offering a novel strategy to combat this lethal malignancy.

The treatment landscape for non-small cell lung cancer (NSCLC) is now well-defined, incorporating a range of modalities such as surgery, chemotherapy, radiotherapy, and immunotherapy. In particular, targeted therapies have gained prominence as first-line treatments for NSCLC patients harboring mutations in genes such as EGFR, ALK, KRAS, HER2, ROS1, and MET, substantially improving survival outcomes. Nevertheless, both innate and acquired resistance to these agents remains a major challenge. Consequently, identifying novel therapeutic targets and strategies continues to be a pressing priority in NSCLC clinical practice. In this study, we present initial evidence establishing druggable RORγ as a key regulator not only of tumor growth but also of metastasis and invasion in NSCLC. Both genetic and pharmacological suppression of RORγ markedly reduced cancer cell viability and invasive behavior. Notably, an RORγ antagonist strongly suppressed the growth and metastatic spread of NSCLC xenograft tumors in murine models. These data highlight RORγ as a promising therapeutic target in NSCLC.

Our RNA-seq gene expression profiling and functional studies revealed that RORγ enhances NSCLC cell growth and invasion by activating Nerve growth factor (NGF) signaling. Specifically, our data demonstrated that RORγ directly binds to the NGF promoter, thereby activating its transcription in NSCLC cells. Beyond direct promoter binding, the transcriptional activity of RORγ is likely modulated by the recruitment of specific co-activators such as nuclear receptor coactivator (NCOA, also known as steroid receptor coactivator (SRC)) family members [12, 15], a key area for future investigation. Furthermore, crosstalk with other transcription factors, such as those of the forkhead box O (FOXO) family (e.g., FOXO1 [16] or FOXO3 [17]), which may form cooperative or antagonistic complexes with RORγ to modulate the NGF axis. Exploring these interactions will be crucial to fully understand the transcriptional network governing NGF expression in NSCLC.

While NGF was initially recognized as the first discovered and extensively studied member of the neurotrophin (NT) family, playing a critical role in the growth and differentiation of various neurons, including both central and peripheral neurons [18, 19], recent studies have revealed its involvement in the occurrence, progression, and metastasis of various tumors through both neural and non-neural mechanisms. Interestingly, NGF acts paradoxically, functioning either as an oncogene or a tumor suppressor, depending on the specific cellular and tissue context. For instance, it serves as an oncogene in osteosarcoma [20], prostate cancer [21] and breast cancer [22, 23], yet exhibits tumor suppressor activity in neuroendocrine small cell lung cancer tumors [24, 25]. Our experimental results further showed that NGF knockdown reduced ERK phosphorylation and significantly inhibited the proliferation, migration, and invasion of NSCLC cells. Conversely, overexpression of NGF or the addition of exogenous NGF potently reversed the inhibitory effects of the RORγ antagonist on NSCLC cell growth, migration, and invasion. these findings establish the RORγ-NGF axis as a pivotal regulator in NSCLC progression.

In summary, our study revealed that RORγ acts as a key determinant of NGF gene expression and is a promising therapeutic target for NSCLC. Given that several clinical-grade RORγ inverse agonists are already available, our results suggest promising translational potential for the development of new treatment avenues in NSCLC. However, given the high sequence homology between RORγ and the immune-specific isoform RORγt, the tools used here—including antagonists W6134 and XY018 and detection antibodies—likely recognize both isoforms. Thus, the effects observed in our immune-competent in vivo models may reflect combined activity in tumor and immune cells, highlighting the need for isoform-selective agents to precisely dissect their functions in the tumor microenvironment. Furthermore, clinical translation must address potential on-target toxicities arising from RORγ’s roles in Th17 immunity and metabolism. Strategies such as tissue-selective delivery or optimized dosing schedules could help mitigate these risks and improve the therapeutic window in future development.

## Supplementary Information

Supplementary Material 1.

Supplementary Material 2.

## Data Availability

All associated data supporting this study are available upon request from the corresponding authors. Gene expression data from RNA-seq analysis were deposited in the NCBI GEO database (GSE308540).

## Abbreviations

NSCLC: Non-small cell lung cancer
RORγ: Retinoic acid receptor-related orphan receptor gamma
NGF: Nerve growth factor
Th17: T helper 17 cells
p-ERK: phosphorylated ERK
IHC: Immunohistochemistry
SPF: Specific pathogen-free
CDX: Cell line-derived xenograft
LUAD: Lung adenocarcinoma
DEGs: Differentially expressed genes
ChIP-seq: Chromatin immunoprecipitation sequencing
NCOA: Nuclear receptor coactivator
SRC: Steroid receptor coactivator
FOXO: Forkhead box O
NT: Neurotrophin
