# Supplementary material for: RORγ drives non-small cell lung cancer progression by upregulating the NGF signaling
Source: Respir Res. 2026 Jan 31;27:101. doi: 10.1186/s12931-026-03523-7 (PMC12947509; doi:10.1186/s12931-026-03523-7)
Supplement: Supplementary file 1 — Supplementary Material 1. [file 12931_2026_3523_MOESM1_ESM.docx]

**Supplementary Material**

**RORγ drives non-small cell lung cancer progression by upregulating the NGF signaling**

Yechun Zeng^1^**^#^**, Guodi Cai^1^**^#^**, Jian Zhang^2^**^#^**, Zhenhua Zhang^1^, Wenxin Yin^3^, Tianmiao Ou^1^, Meng Xu^4^, Jing Li^5^, Zhanfang Kang^6^, Junguo Bu^7^, Junjian Wang^1,8^*****, Jie Huang^9^*****, Weineng Feng^4^*****

**Supplementary Table S1**

| **Sequences for siRNA** |  |
| --- | --- |
| siControl | CAGTCGCGTTTGCGACTGG |
| siRORC#1 | CGAGGATGAGATTGCCCTCTA |
| siRORC#2 | GCCCTCATATTCCAACAACTT |
| siNGF#1 | CAACAGTGTATTCAAACAGTA |
| siNGF#2 | GCGGTCATCATCCCATCCCAT |

**Supplementary Table S2**

| **Primers for qPCR** |  |
| --- | --- |
| β-actin F | GAGAAAATCTGGCACCACACC |
| β-actin R | ATACCCCTCGTAGATGGGCAC |
| NGF F | ACCCGCAACATTACTGTGGACC |
| NGF R | GACCTCGAAGTCCAGATCCTGA |

**Supplementary Table S3**

| **Primers for ChIP-qPCR** |  |
| --- | --- |
| ChIP-NGF-F | TCACTTGCGCGTTATCCACT |
| ChIP-NGF-R | GAAATGTCCCGAGTGGGTGT |
