# Supplementary figures and images for: RORγ drives non-small cell lung cancer progression by upregulating the NGF signaling
Source: Respir Res. 2026 Jan 31;27:101. doi: 10.1186/s12931-026-03523-7 (PMC12947509; doi:10.1186/s12931-026-03523-7)

Figure 1C

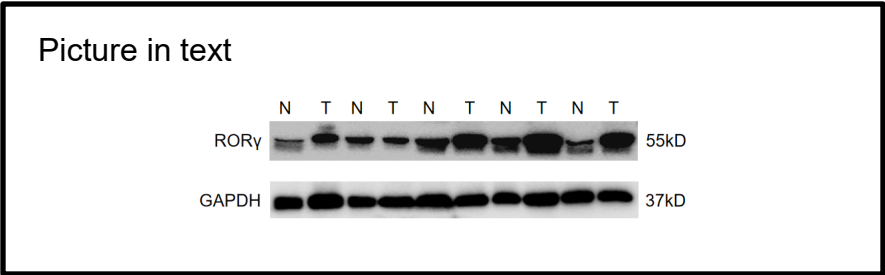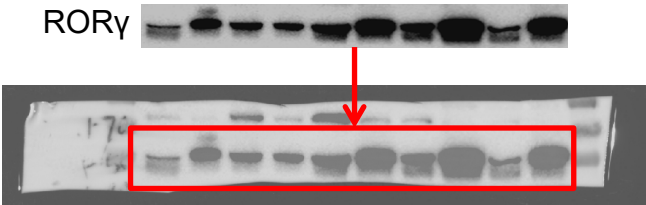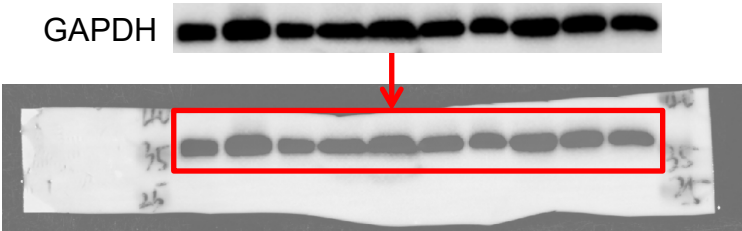

Figure 2A

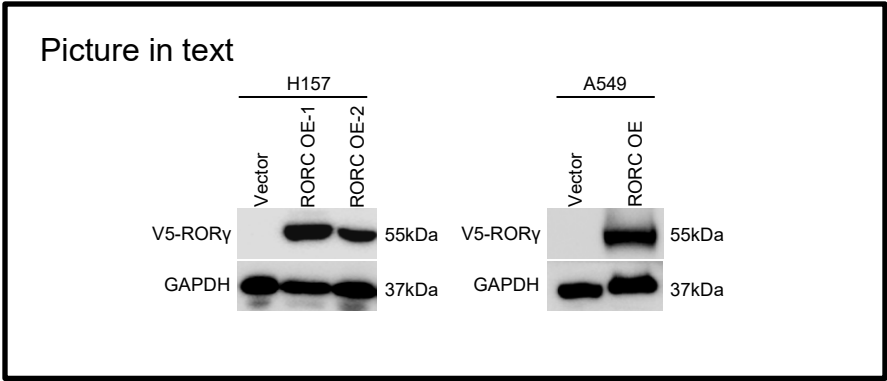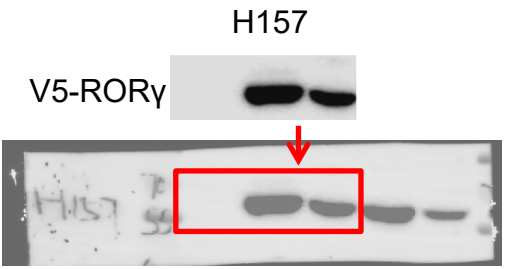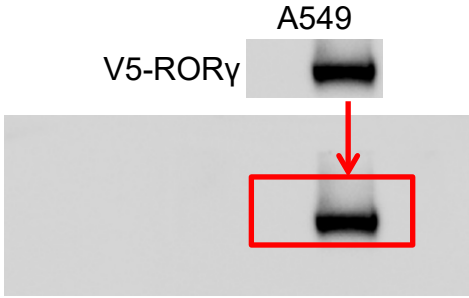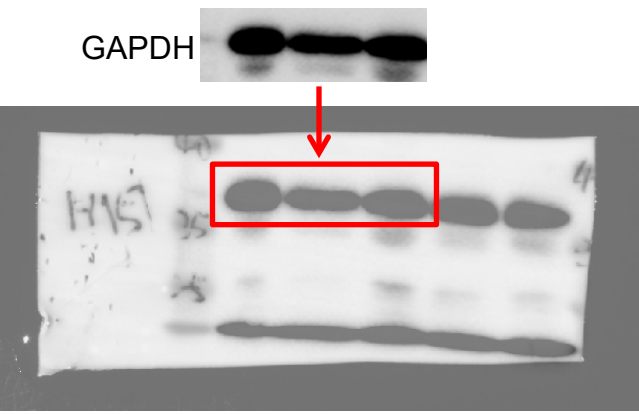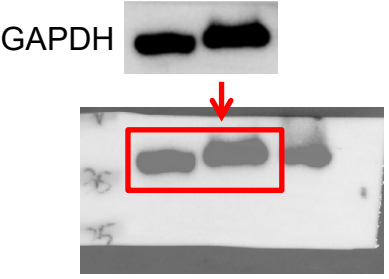

Figure 3C

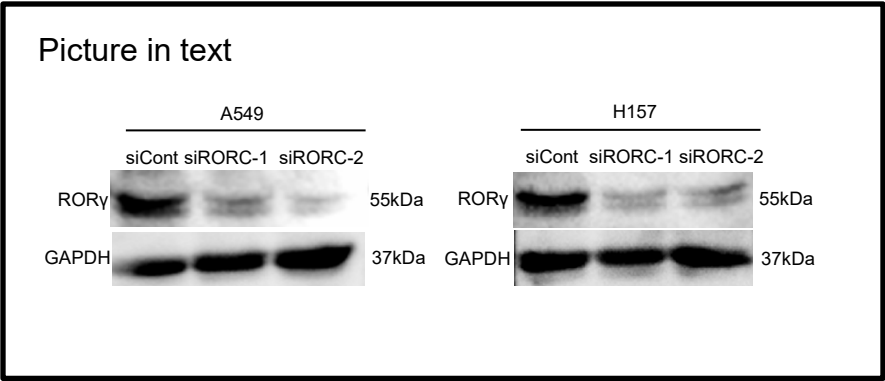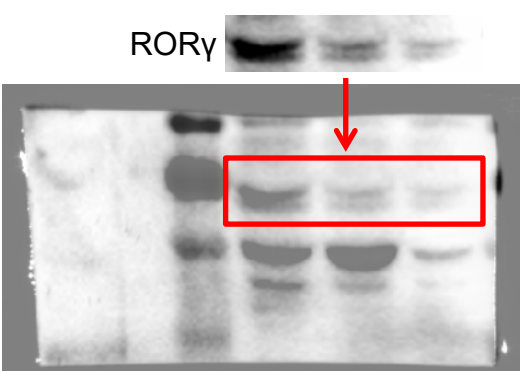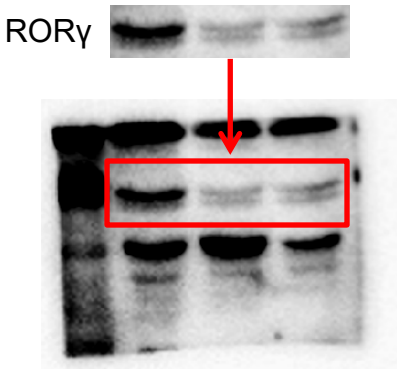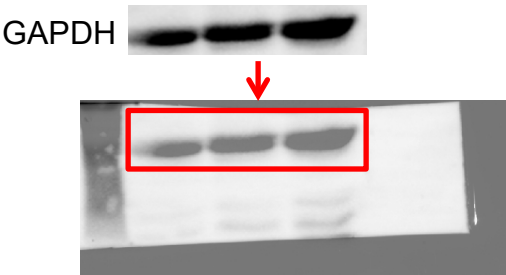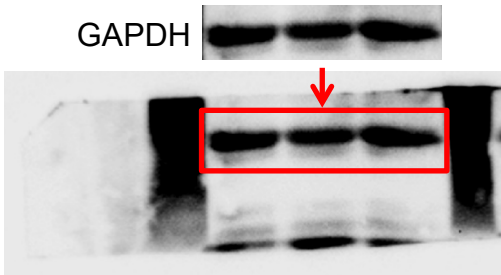

Figure 3C

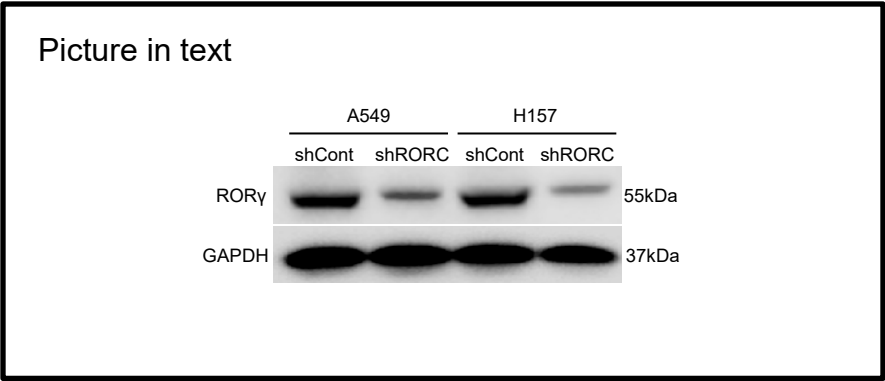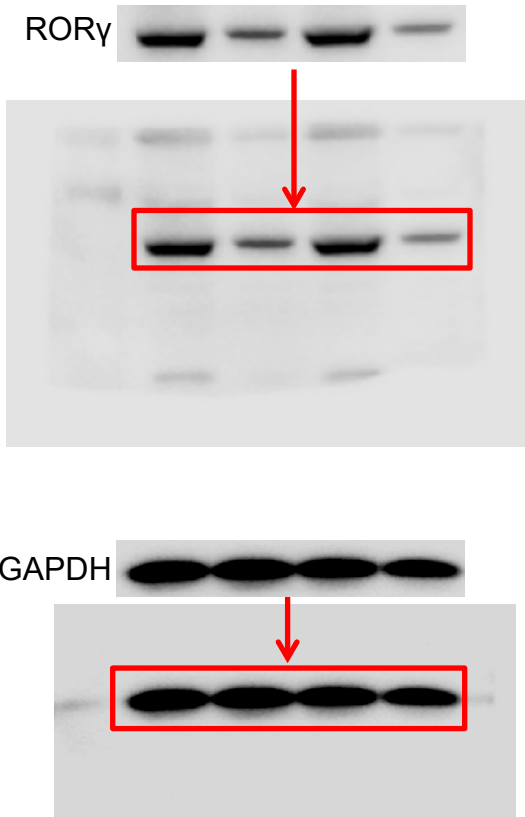

Figure 4C

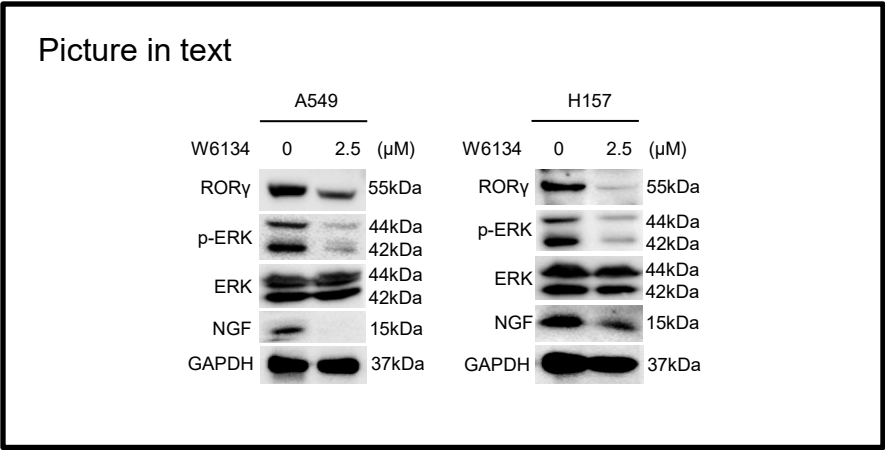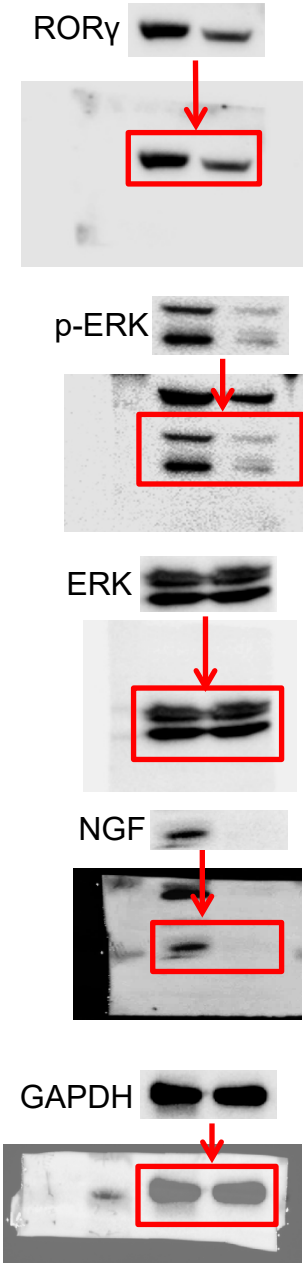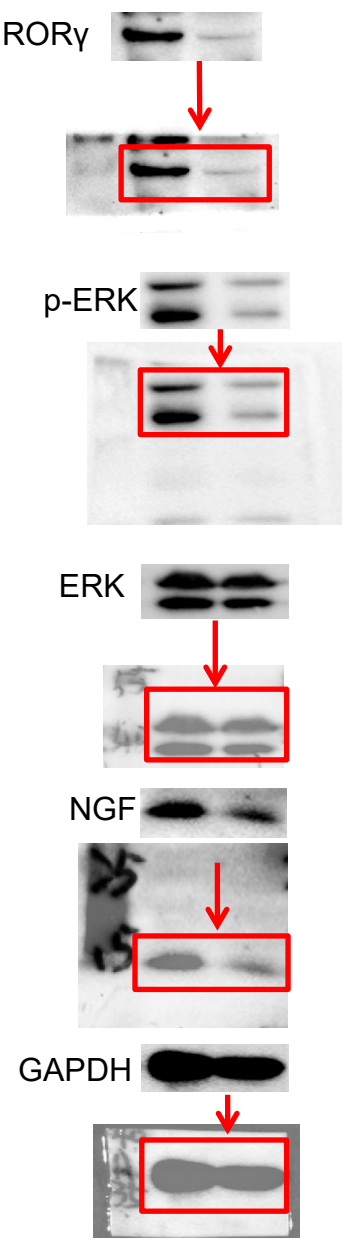

Figure 5

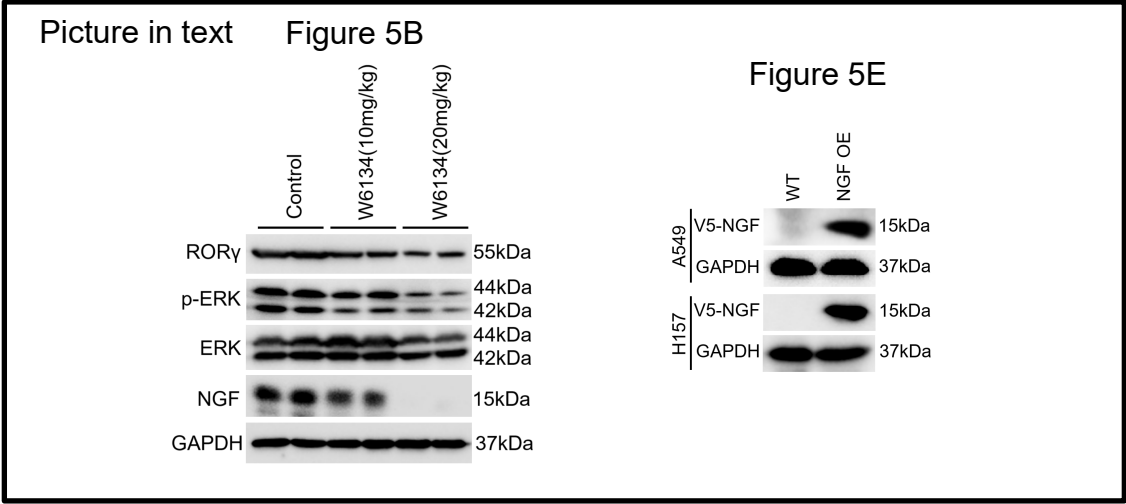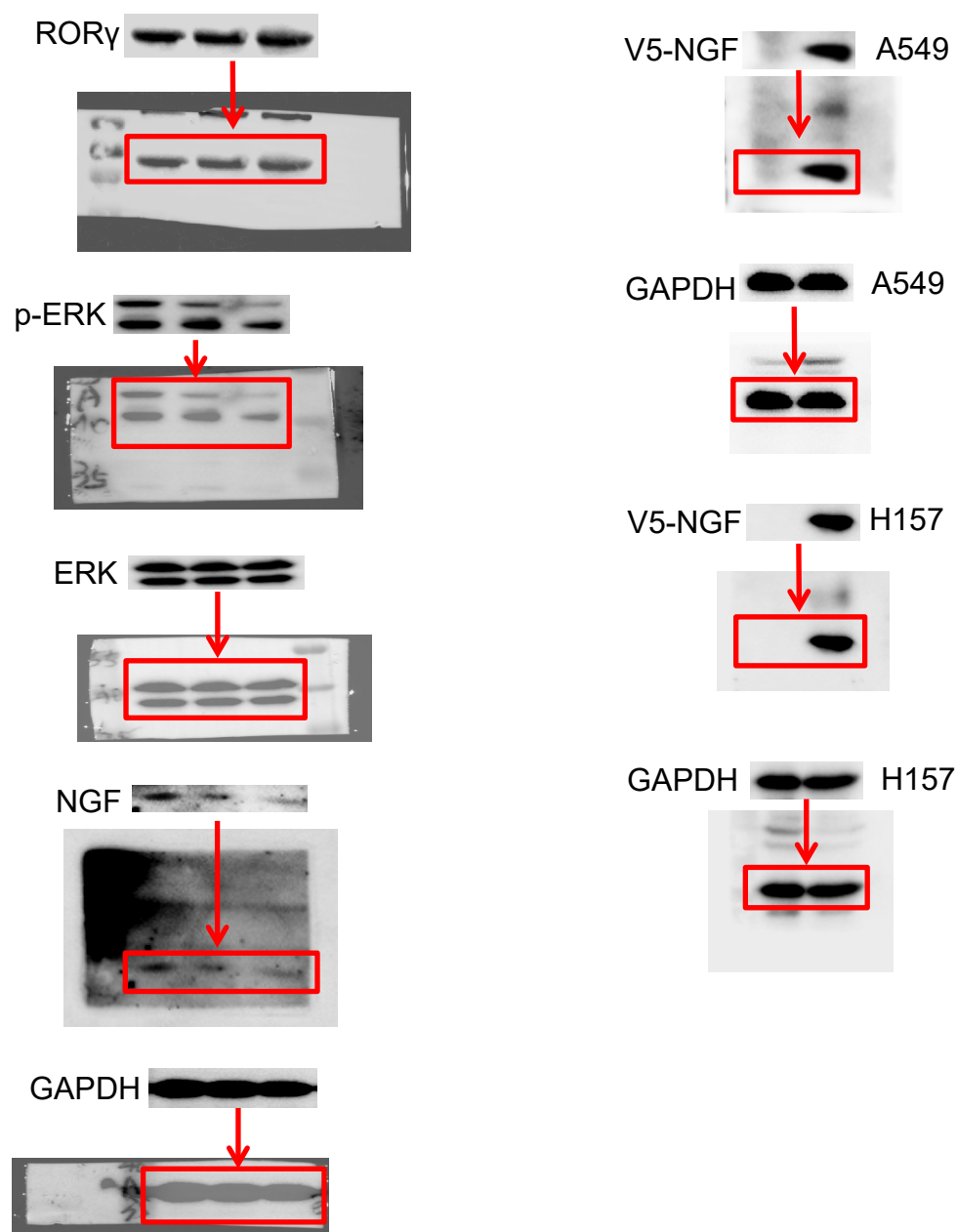

Figure 6K

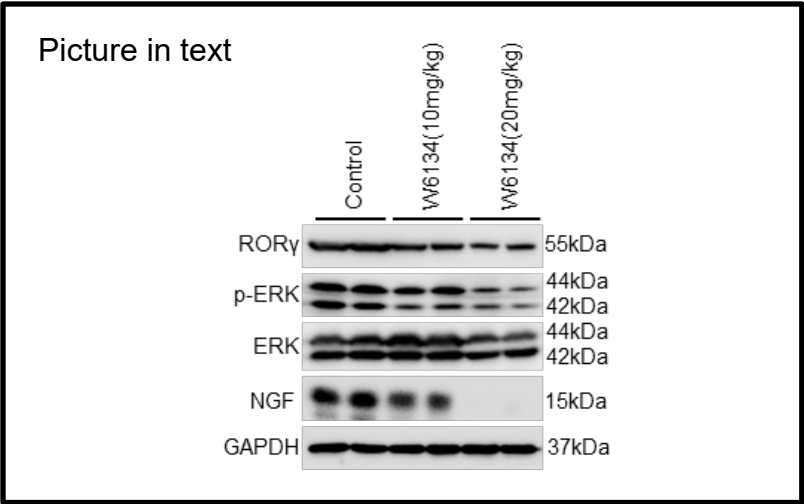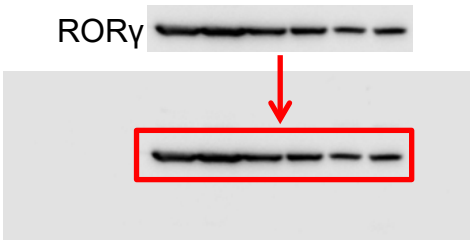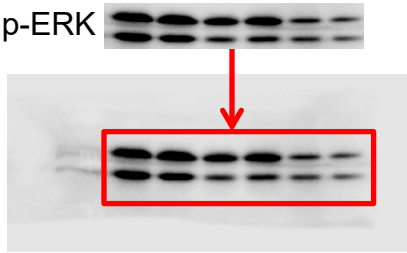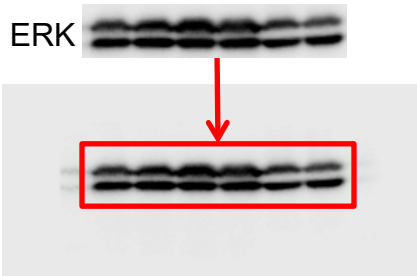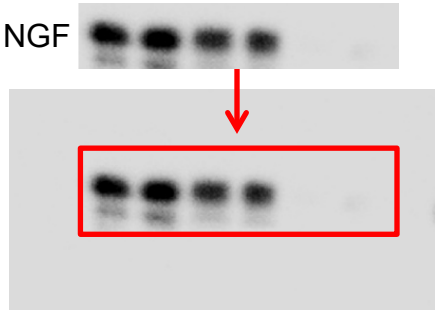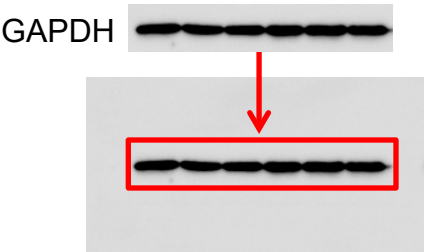

Supplement: Supplementary file 2 — Supplementary Material 2. [file 12931_2026_3523_MOESM2_ESM.pdf]
